# Non-invasive environmental DNA sampling reveals tuberculosis risks at the human – Great Ape Interface in Africa

**DOI:** 10.1080/22221751.2026.2645874

**Published:** 2026-03-23

**Authors:** Ernest Kalalizi, Luis Flores, Marta Pérez-Sancho, Alberto Perelló, Carmen Herranz, Laura Herrera, Beatriz Romero, Prince Kaleme, Teresa García-Seco, Déo Kujirakwinja, Arthur Kalonji, Zacharie Kashongwe, Freddy Birembano-Machara, Daniel Baganda, Pacific Nkonzi, Itsaso Vélez Del Burgo, Frederic Le Gal, José De La Fuente, Lucas Domínguez, Christian Gortázar

**Affiliations:** a SaBio Instituto de Investigación en Recursos Cinegéticos (IREC) CSIC-UCLM-JCCM, Ciudad Real, Spain; b L'Université Cinquantenaire de Lwiro, South Kivu, Democratic Republic of Congo; c Lwiro Primate Rehabilitation Center (LPRC), South Kivu, Democratic Republic of Congo; d Centre de Recherche en Sciences Naturelles de Lwiro (CRSN Lwiro), South Kivu, Democratic Republic of Congo; e One Health Conservation Initiative (OHCI), Lwiro, Democratic Republic of Congo; f Faculty of Veterinary, Department of Animal Health, VISAVET Health Surveillance Center, Complutense University of Madrid, Madrid, Spain; g Faculty of Veterinary, Department of Animal Health, Complutense University of Madrid, Madrid, Spain; h Department of Bacteriology, National Centre of Microbiology, Instituto de Salud Carlos III, Majadahonda, Spain; i Institut Supérieur des Techniques Médicales de Bukavu, South Kivu, Democratic Republic of Congo; j Institut Supérieur de Tourisme, North Kivu, Democratic Republic of Congo; k Kahuzi-Biega National Park, South Kivu, Democratic Republic of Congo; l University of Kinshasa (Unikin), Kinshasa, Democratic Republic of Congo; m Le Programme National de Lutte Contre la Tuberculose (PNLT), Kinsasa, Democratic Republic of Congo; n Laboratoires de Microbiologie Clinique, Bobigny, France; o Department of Veterinary Pathobiology, Center for Veterinary Health Sciences, Oklahoma State University, Stillwater, OK, USA

**Keywords:** Gorilla Beringei Graueri, human-wildlife interface, non-invasive sampling, reverse zoonosis, South Kivu

## Abstract

The current range of African great apes includes countries with some of the world’s highest incidence rates of human tuberculosis (TB). Non-human primates (NHPs) living in their natural habitats are expected to be free of TB. However, TB represents a known threat to captive NHP communities. We applied a non-invasive sponge-based environmental DNA (eDNA) sampling to run a cross-sectional survey at the human-animal interface in a challenging setting: the East of the Democratic Republic of Congo (DRC). The study sites included a primate rehabilitation centre, the local health area, and a nearby national park with critically endangered Eastern Lowland Gorillas (*Gorilla beringei graueri*). Sponge samples were tested for two PCR targets, IS*6110* and *mpb*70. Positive samples were further characterized by spoligotyping, species identification and detection of molecular resistance against rifampicin and isoniazid. We detected *Mycobacterium tuberculosis* eDNA in 26% of the samples from all three sites including samples linked to humans, wild gorillas and captive NHPs. The spoligotype could be identified in 18 cases. Spoligotype SIT130 was detected in all sites including human and gorilla environment samples. These findings are strongly suggestive of epidemiological links between human and NHP TB in equatorial Africa.

## Introduction

There are four great ape species in equatorial Africa: Bonobo (*Pan paniscus*), Chimpanzee (*P. troglodytes*), Eastern Gorilla (*Gorilla beringei*), and Western Gorilla (*G. gorilla*). All are endangered due to habitat loss, disease, poaching and conflict with humans [1,2]. Among Eastern Gorillas, the Eastern Lowland Gorilla (*Gorilla beringei graueri*) is the most critically endangered subspecies. It is endemic to the Democratic Republic of Congo (DRC) and restricted to a few remnant patches of well-preserved rainforest in central eastern DRC, including Kahuzi-Biega National Park (KBNP). Human-great ape contact occurs in zoos and wildlife rehabilitation centres, involving visitors and, especially, caretakers [3], and in the field, involving residents, park rangers and tourists visiting habituated groups, as well as poachers and bushmeat consumers [2,4]. The threat posed to great ape conservation by infectious disease outbreaks is increasing due to habitat loss and rising human pressure [5].

The geographical range of African great apes overlaps with countries that have some of the world's highest rates of human tuberculosis (TB) caused by *Mycobacterium tuberculosis* and closely related members of the *M. tuberculosis* complex (MTBC) [6]. In 2022, the DRC had a human TB incidence rate of 317 per 100,000 inhabitants and a TB mortality rate of 39 per 100,000, with 244,000 cases of tuberculosis and 4,000 deaths [7]. Undernourishment was identified as the main suspected risk factor for human TB, followed by alcohol use disorders and AIDS [7]. The war-torn South Kivu region in the eastern DRC is a disease emergence hotspot at the animal-human interface [8].

The mainstream view on MTBC transmission is that airborne transmission is the primary mode [9]. However, mycobacteria evolved from environmental organisms [10] and even the causative agents of human and animal TB, which are adapted to mammalian hosts, can survive in the environment [11–13]. This suggests that members of the MTBC can be transmitted not only by the respiratory route but also orally. Few studies have successfully attempted to monitor environmental MTBC contamination by culture [12] or by combining flow cytometry, fluorescence *in situ* hybridization, and fluorescence-activated cell sorting [13]. More often, environmental DNA (eDNA)-based methods are used since these are sensitive, time-efficient, affordable, and less affected by contaminating organisms [14–19]. In studies on TB in NHPs, eDNA is less logistically demanding than skin-testing, blood-drawing, x-rays and physical examinations. Thus, molecular detection of MTBC using biological samples that are easy to collect, and store holds promise for advancing our ability to detect and characterize MTBC circulation in NHPs and their environment [20].

**Figure 1.**
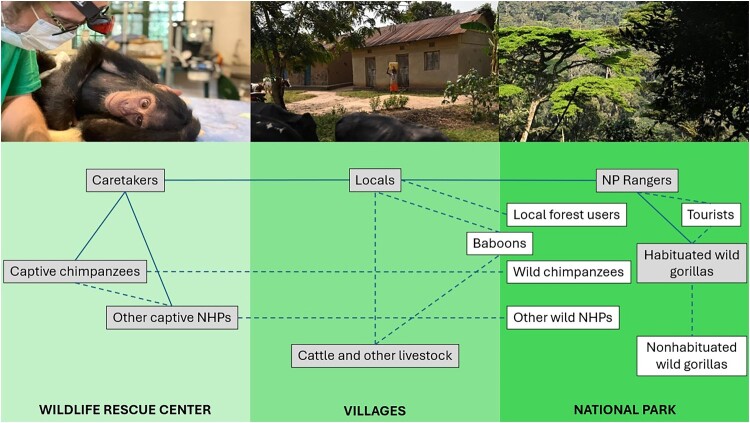
Diagram of the study setting. Infection transmission pathways are based on literature [6,24] and the authors’ experience. Solid connections represent highly likely contact probabilities. Dashed connections represent suspected and indirect ones. Grey background indicates environments or subjects sampled in this study.

Limited scientific information is available on the circulation of MTBC in African NHP rehabilitation centres (Table S1). Generally, NHPs living in their natural habitats are expected to be free of TB [21,22]. Only one single case of natural MTBC infection was recorded in a free-ranging great ape, a chimpanzee [23]. Furthermore, the risk posed by humans to great ape conservation is often mentioned [24] but information on actual MTBC detection is not available. We applied a newly developed non-invasive sponge-based eDNA collection protocol and tested two PCR targets, IS*6110* and *mpb*70, in an epidemiologically challenging setting in eastern DRC: a primate rehabilitation centre hosting nine NHP species, the local health area and hospital, and a nearby national park with human-habituated eastern lowland gorillas (Figure 1).

Positive samples were further characterized by spoligotyping, real-time PCR to identify the *Mycobacterium* species and identification of mutations within the *rpo*B, *inh*A and *kat*G genes to detect resistance against rifampicin and isoniazid. We detected eDNA MTBC markers in wild and captive primates, and their environment, and identified epidemiological links to in-contact humans.

## Materials and methods

### Ethical approval

This study was approved by the Institutional Health Ethics Committee (CIES) of the Catholic University of Bukavu under Decision No. UCB/CIES/NC/020/2023. Human samples were collected after explaining the study in the participants’ local language, and verbal consent was obtained from all participants due to their low level of literacy. Some results from this study are part of the TTHALESS project (Transmission de la Tuberculose Humaine et Animale à Lwiro: Études des Souches de Mycobactéries), which was also approved by the Institutional Health Ethics Committee (CIES) of the Catholic University of Bukavu, under Decision No. UCB/CIES/NC/016/2022.

### Study sites

This cross-sectional survey was conducted in the Miti-Murhesa health zone, one of the 30 health zones in South Kivu province, Eastern DRC. In this health zone, we find the Lwiro Primate Rehabilitation Centre (LPRC), the highland of Kahuzi-Biega National Park (KBNP), and Lwiro village (LV), the three sites where the sample collection and processing for the study took place.

The KBNP was established in 1970 to protect the Eastern Lowland or Grauer's Gorilla, the largest gorilla species. Kahuzi Biega National Park is the only accessible destination with many Eastern Lowland Gorillas, with more than 400 individuals, including seven habituated groups.

The LPRC is located on the land of the Centre de Recherche en Sciences Naturelles de Lwiro (CRSN Lwiro). The LPRC is a centre under the management of the Institute Congolais pour la Conservation de la Nature (ICCN), where animals seized by ICCN are brought in for rehabilitation. During the study, the LPRC housed 121 chimpanzees, divided into seven groups: Baby Chimps Quarantine House (BCQH, n = 12); Baby Chimps Quarantine Group (BCQG, n = 11); Young Chimps Sanctuary Group (YCSG, n = 11); Pori Chimps Group (PCG, n = 33); Big Male Group (BMG, n = 7); Watoto Chimps Group (WCG, n = 21); and Kiyana Chimps Group (KCG, n = 26). Additionally, the centre housed 120 other non-human primates (NHPs) of 14 different species, distributed into various groups: Baboon Group (BG) with Olive Baboons (*Papio anubis*, n = 21) and Yellow Baboons (*Papio cynocephalus*, n = 2); Blue Monkey Group (*Cercopithecus mitis*, CMG, n = 12); *C. ascanius* Group (CAG) with De Brazza's Monkey (*C. neglectus*, n = 2); Mona Monkey (*C. denti*, n = 1); and *C. ascanius* (n = 16); *C. l´hoesti* Group (CLG) with L'Hoesti's Monkey (n = 9); *C. hamlyni* Group (CHG) with Owl-faced Monkey (n = 15); Mangabey Group (MG) with Agilis Mangabey (*Cercocebus agilis*, n = 1); Grey-cheeked Mangabey (*Lophocebus albigena*, n = 3) and Patas monkey (*Erythrocebus patas*, n = 1); Vervet Group (VG) with different species of green monkeys (*Chlorocebus aethiops*, n = 11; *C. cynosures*, n = 11; *C. tantalus*, n = 3); and the *Colobus guereza occidentalis* group (CGG), (n = 12). The centre is managed by a team of 58 workers who care for the primates under the supervision of a manager.

Lwiro Hospital (LH), located in LV, belongs to the Nutrition Department of the Centre de Recherche en Sciences Naturelles de Lwiro (CRSN Lwiro). It provides essential healthcare services to the surrounding communities, including those involved in conservation and research at the nearby CRNS Lwiro and the LPRC. The hospital plays a critical role in addressing public health needs in the region, including treating common diseases such as malaria, TB, and other infectious diseases. It also serves as a key medical facility for both residents and those working in conservation efforts within the region, including the KBNP. Lwiro Hospital is the place where specialized human TB consultations were conducted during the first phase of the TTHALESS project.

### Environmental DNA sampling

From October 2023 to May 2024, 178 pre-hydrated environmental DNA (eDNA) sampling sponges (GPSponge®, Genetic PCR Solutions, Alicante, Spain) containing 15 mL of an isotonic surfactant nucleic acid-preserving liquid [19] were deployed on surfaces in KBNP (n = 39), LPRC (n = 116), and the Miti-Murhesa health area including LH (n = 23). Sample sizes are summarized in Table 1. The samples taken included, in the KBNP, Gorilla faeces, n = 11; Gorilla nests, n = 10; Gorilla leftover food, n = 10; Personal Protection Equipment (PPE), n = 3; Human body surfaces: n = 5. In LPRC: samples from body surfaces of anesthetized non-human primates (NHPs), n = 11 including 7 Chimpanzees, 2 Olive Baboons, 1 Blue Monkey and 1 Black-and-white Colobus; Human body surfaces, n = 5; Indoor leftover food, n = 6; Leftover food in feeders, n = 11; NHP faeces, n = 8; NHP enclosure surfaces, n = 41; outer facility surfaces, n = 32; and personal protection equipment (PPE), n = 2. In the Miti-Murhesa health area and LH: Cattle milk and faeces (n = 6) and human body surfaces, n = 15, and 2 consultation room surfaces. The patients from LH were cases of active TB that had been diagnosed by GenExpert PCR from sputum and stool samples in the TTHALESS study (Table S2).

**Table 1.** Sample size by type, site and category.

Sample type	Site	N	Category
Human body surface	LPRC	5	Human
Body (anesthetized NHP)	LPRC	11	Primate
NHP faeces	LPRC	8	Primate
Indoor leftover food	LPRC	6	Environment
NHP feeders with food	LPRC	11	Environment
NHP enclosures	LPRC	41	Environment
NHP facility surfaces	LPRC	32	Environment
PPE	LPRC	2	Human
Human body surface	LH	15	Human
Hospital surfaces	LH	2	Environment
Cattle faeces	LV	4	Livestock
Cattle milk	LV	2	Livestock
Human body surface	KBNP	5	Human
Gorilla faeces	KBNP	11	Primate
Gorilla nests	KBNP	10	Primate
Gorilla leftover food	KBNP	10	Environment
PPE	KBNP	3	Human
**Total**		**178**	** **

NHP non-human primate, PPE personal protective equipment, LPRC Lwiro Primate Rehabilitation Centre, LH Lwiro Hospital, LV Lwiro village, KBNP Kahuzi-Biega National Park.

During sampling, the sponge was gently rubbed 5 times on each sampling site to facilitate the collection of nucleic acids present in the environment [25]. All samples were collected by the same researcher (EK) and stored at −20°C for 3–5 days before liquid extraction. The final volume before DNA extraction was around 14 mL.

### DNA extraction

DNA was extracted using the QIAamp® Fast DNA Stool Mini Kit (QIAGEN, Hilden, Germany). Briefly, 15 µL of proteinase K was added to each Eppendorf tube before adding 200 µL of our sample, followed by 200 µL of AL buffer, which was vortexed for 15 s and incubated at 95°C for 30 min. Once finished, 200 µl of 96% ethanol was added to the lysate. Subsequently, 600 uL of lysate was transferred to a QIAamp spin column provided with the kit and centrifugation was carried out at 13000 rpm for 1 min. Then the collection tube was discarded and replaced by a new one. In the following step, 500 µL of AW1 buffer was added to the column under centrifugation at 13,000 rpm for 1 min and then the collection tube was discarded and replaced by a new tube for the second time. In the following step, 500 µL of AW2 buffer was added and then centrifuged at 13,000 rpm for 3 min until the column membrane was dry. The collection tube was discarded followed by the replacement of another new tube for the third time. The column content was placed in a new 1.5 mL Eppendorf tube and 100 µL of pre-warmed ATE buffer was added, incubating for one min at room temperature. Finally, the centrifugation was done at 13,000 rpm for 1 min. Extraction controls were used to ensure contamination-free protocols. The purified DNA was stored at -40°C until PCR analysis.

### PCR primers and protocol

We defined two real-time PCR targets to detect MTBC DNA in environmental samples, IS*6110* and *mpb*70. The primer sequences for the IS*6110* multicopy target were *6110*-forward: 5´-GGTAGCAGACCTCACCTATGTGT-3´, *6110*-reverse: 5´-AGGCGTCGGTGACAAAGG-3´ and *6110*-probe: 5´-FAM-CACGTAGGC GAACCC – MGB NFQ-3´ [26]. The primer sequences for the *mpb*70 monocopy target were *mpb*70 - forward: 5′-CTCAATCCGCAAGTAAACC-3′, *mpb*70-reverse: 5′-TCAGCAGTGACGAATTGG-3′ and *mpb*70-probe: 5′-FAM CTCAACAGCGGTCAGTACACGGT-BHQ1-3′ [27].

PCR was performed using the QuantiFast Pathogen + IC Kits (400) according to the manufacturer's instructions (QIAGEN). Each reaction included an internal amplification control (IAC) to detect PCR inhibition, while ultra-pure distilled water and DNA from a BCG inoculum were used as negative and positive controls, respectively, to validate each PCR reaction. All PCR reactions were performed in a CFX96 TouchTM real-time PCR detection system (Bio-Rad, Hercules, CA, USA) under the following cycling conditions: 95°C for 5 min, followed by 45 2-step cycles of 95°C for 15 s and 60°C for 1 min.

The Ct thresholds were 38 and 40 for IS*6110* and *mpb*70, respectively. A sample was considered negative when both markers were negative, and 6 samples were considered inhibited due to no amplification of the IAC in PCR reaction. All PCR procedures were performed under controlled laboratory conditions to minimize the risk of contamination. Preparation of the PCR reagent mix was carried out in a clean area, while the addition of sample DNA was performed in an intermediate area. Amplification in the thermocycler was conducted in a separate dirty area. This spatial separation of pre- and post-amplification steps ensured the reliability of the results throughout the process.

### Spoligotyping, MTBC species identification and molecular resistance

All PCR positive samples were characterized by DVR-spoligotyping as previously described [28] but only samples with IS*6110* PCR Ct values <33 showed readable profiles. Spoligotyping profiles were assigned according to the SIVIT2 database [29]. A commercial RT–PCR kit was used to detect the species *M. tuberculosis*, *M. africanum*, *M. microti*, *M. caprae*, *M. bovis*, *M. bovis BCG* and *M. canettii* following the instructions specified by the manufacturer (Vircell Microbiologist, Granada, Spain). The Fluorotype MTBDR version 2.0 (Hain Lifescience, Nehren, Germany) was used to detect resistance to rifampicin and isoniazid.

Spoligotyping epidemiological links were represented using network approaches to describe associations among pathogen genotypes, and host species. A weighted, undirected bipartite network was constructed linking each spoligotype to the host species in which it was detected. Nodes represent categories (spoligotypes, or species) and edges represent observed detections, with edge weights corresponding to the number of samples supporting each association. Networks were constructed and analyzed in R software version 4.4.1. using the *igraph* package version 2.1.4.

Chi-square tests of independence were performed using R software version 4.4.1. to examine the association between sampling site and the positivity of IS*6110* and *mpb*70 molecular markers. Effect sizes were quantified using Cohen's w. Statistical power was assessed through two complementary approaches using GPower 3.1.9.7; a sensitivity analysis to determine the minimum detectable effect size given the sample size (n = 178), desired power (1-β = 0.95), and significance level (α = 0.05); and a post-hoc power analysis to evaluate the achieved statistical power given the observed effect sizes and the number of complete cases (n = 172; not-inhibited samples).

## Results

### Detection of *Mycobacterium tuberculosis* complex eDNA

We detected MTBC eDNA in sponge samples collected in the Lwiro Primate Rehabilitation Centre (LPRC), the highland of Kahuzi-Biega National Park (KBNP), and the Miti-Murhesa health area (local cattle and Lwiro Hospital, LH) including samples linked to humans, cattle samples, samples collected on wild gorilla faeces, and samples linked to kept NHPs. We tested a total of 178 sponge samples. Of these, 6 NHP facility surface samples from the LPRC yielded no PCR result due to polymerase inhibitors. Of the 172 remaining samples, 61 tested positive for IS*6110* (35.47%; 95% CI 28.24–42.69%; CT range 22–37) and 33 for *mpb*70 (19.19%; 95% CI 13.24–25.13%; CT range 26–38). All but one *mpb*70 positive samples (32/33, 97%) were also IS*6110* positive. Thus, 32 samples (18.60%) tested positive for both markers. Considering positivity to any marker, the mean prevalence was 62 of 172, (36.05%, 95% CI 28.79–43.29%). Table 2 shows the overall results by sample type and site, and Table 3 indicates the prevalence per sampling site.

**Table 2.** M. tuberculosis complex PCR results in 165 environmental sponge samples from eastern DR Congo, by site and sample type.

Sample type	Site	N	Inh.	IS*6110*	*mpb*70	Pos. to both	Neg. to both
Human body surface	LPRC	5	0	3	3	3	2
Body (anesthetized NHP)	LPRC	11	0	3	2	2	8
NHP faeces	LPRC	8	0	3	3	2	4
Indoor leftover food	LPRC	6	0	1	0	0	5
NHP feeders with food	LPRC	11	0	2	1	1	9
NHP enclosures	LPRC	41	5	9	3	3	27
NHP facility surfaces	LPRC	32	1	8	4	4	23
PPE	LPRC	2	0	0	0	0	2
Human body surface	LH	15	0	14	11	11	1
Hospital surfaces	LH	2	0	1	0	0	1
Cattle faeces	LV	4	0	4	0	0	0
Cattle milk	LV	2	0	1	0	0	1
Human body surface	KBNP	5	0	3	2	2	2
Gorilla faeces	KBNP	11	0	6	4	4	5
Gorilla nests	KBNP	10	0	2	0	0	8
Gorilla leftover food	KBNP	10	0	0	0	0	10
PPE	KBNP	3	0	1	0	0	2
**Total**	** **	**178**	**6**	**61**	**33**	**32**	**110**

NHP non-human primate, PPE personal protective equipment, LPRC Lwiro Primate Rehabilitation Centre, LH Lwiro Hospital, LV Lwiro village, KBNP Kahuzi-Biega National Park, Inh. =  PCR reaction inhibited for both markers.

**Table 3.** Detection prevalence of the IS6110 and mpb70 molecular markers of *M. tuberculosis* complex in 165 environmental sponge samples from eastern DR Congo, by sampling site, with 95% confidence intervals (95% CI). Prevalence estimates were calculated excluding the six inhibited samples.

Sampling site	Molecular marker	Prevalence (%)	Lower 95% CI	Upper 95% CI
LPRC	IS*6110*	26.4	19.0	35.3
	*mpb*70	14.5	9.2	22.3
LH	IS*6110*	88.2	65.7	96.7
	*mpb*70	64.7	41.3	82.7
LV	IS*6110*	83.3	43.6	97.0
	*mpb*70	0.0	0.0	39.0
KBNP	IS*6110*	30.8	18.6	46.4
	*mpb*70	15.4	7.2	29.7

LPRC Lwiro Primate Rehabilitation Centre, LH Lwiro Hospital, LV Lwiro village, KBNP Kahuzi-Biega National Park.

In the LPRC, we detected 15 of 110 valid (non-inhibited) samples (13.6%) positive for both markers, including human body surface samples (n = 3), anesthetized NHP surface samples (n = 2; 1 chimpanzee and 1 olive baboon), a feeder in the baboon enclosure; NHP faecal samples from two different chimpanzee enclosures (n = 2) and the colobus enclosure (n = 1), chimpanzee facility surface samples (n = 2), and several (n = 4) surface samples from other LPRC facilities: a medical jar, freezer handles, a wash basin and post-mortem room surfaces.

In the LH of the Miti-Murhesa health area we tested 15 human body surface samples and 2 facility surface samples. We found 11 of the former positives for both markers. Three additional patient surface samples tested positive for IS*6110* only. All 15 corresponded to patients included in the TTHALESS project who presented symptoms compatible with active TB and attended consultation. Regarding cattle from the Miti-Murhesa health area, all four faecal samples tested positive for IS*6110*. Additionally, one of the two milk samples was also positive for the same marker.

In KBNP both PCR targets, IS*6110* and *mpb*70, were positive in 2 of 5 samples taken from KBNP rangers and 4 of 11 sponge samples taken from gorilla faeces. Another 6 KBNP samples were positive only for IS*6110* including one additional ranger, two gorilla faecal sponge samples, 2 of 10 gorilla nest sponge samples, and one PPE sponge sample taken from a ranger’s boot after visiting habituated gorillas for sampling. In total, 12 of 39 (30.8%) samples taken in KBNP tested positive for at least one of the PCR targets. Taken together, these results evidence a high prevalence of human and NHP contact with MTBC eDNA in the study sites.

Chi-square tests of independence revealed significant associations between sampling site and both molecular markers: IS*6110* (χ² = 31.05, df = 3, *p* < 0.001) and *mpb*70 (χ² = 26.03, df = 3, *p* < 0.001). Effect sizes were medium-to-large according to Cohen's criteria (w = 0.42 for IS*6110* and w = 0.39 for *mpb*70). Sensitivity power analysis indicated that, with 178 samples and α = 0.05, the study had 95% power to detect effects as small as w = 0.31. Given the observed effect sizes (w = 0.42 and w = 0.39), post-hoc power analysis confirmed >99% statistical power for both markers (99.88% for IS*6110* and 99.49% for *mpb*70).

### Species identification, spoligotyping and molecular resistance

Of the 61 IS*6110* positive samples, 43 (70.5%) were further confirmed as belonging to the MTBC by spoligotyping (n = 43), FluoroType MTBDR (n = 30) or Vircell (n = 17), including all IS*6110* + *mpb*70 positive samples and 11 of 29 (38%) IS*6110* positive but *mpb*70 negative samples. In all 20 cases where a species ID was possible, the MTBC species identified was *M. tuberculosis* sensu stricto.

**Figure 2.**
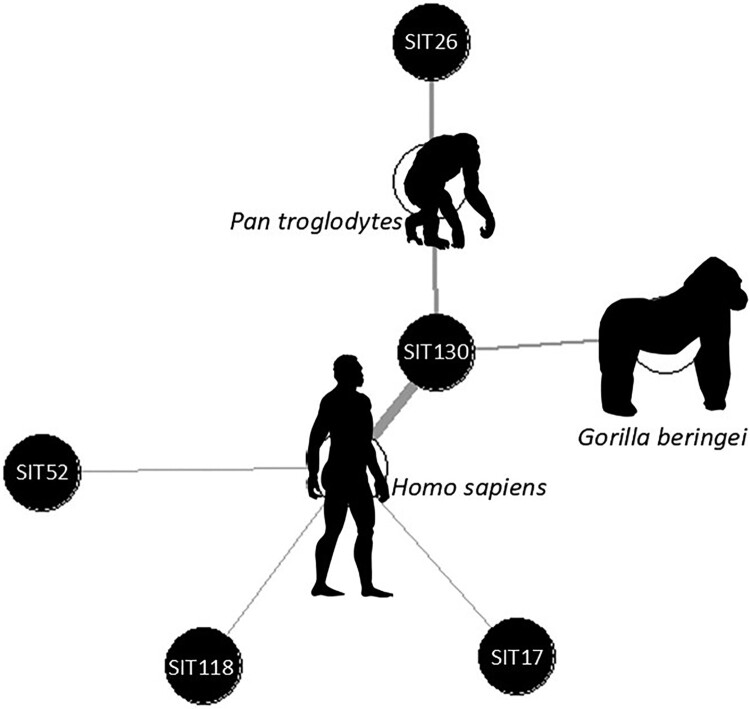
Spoligotype-species network representation. The spoligotype pattern could be identified in 18 samples. Three samples belonged to spoligotype SIT26, only detected in chimpanzees, while 12 samples belonged to spoligotype SIT130, which constitute the spoligotype shared between species. Three samples belonged to spoligotypes SIT17, SIT52, and SIT118, respectively, only detected in humans.

The spoligotype pattern could be identified in 18 samples. Three samples belonged to spoligotype SIT26, while 12 samples belonged to spoligotype SIT130. Three samples belonged to spoligotypes SIT17, SIT52, and SIT118, respectively. Seven additional samples were suspected of hosting dual infections, but precise identification was not possible. The three SIT26-positive samples were faecal samples from two different LPRC chimpanzee groups (WCG and YCSG) and a floor sample taken from a third chimpanzee group (KCG). Spoligotype SIT30 was detected in all study sites, including human and NHP linked samples from LPRC and KBNP. See Figure 2 for spoligotype-species network.

In KBNP, 10 of 11 PCR positive samples were confirmed as belonging to the MTBC by spoligotyping or FluoroType MTBDR. Furthermore, 2 human surface samples and 2 gorilla faecal samples were confirmed to belong to the species *M. tuberculosis*, 3 of them with the pattern SIT130. This is the first MTBC eDNA detection in free ranging gorillas and the first evidence suggesting contact of humans and free ranging NHPs with the same spoligotype pattern.

Of the 30 samples in which FluoroType detected MTBC, none showed mutations in the *rpo*B, *inh*A, or *kat*G genes, and therefore, no resistance to rifampicin or isoniazid was detected.

### Sponge-sample eDNA PCR sensitivity and sampling targets

Human Body Surface samples from LH yielded the highest positivity rate, 14/15 (93.3%) for IS*6110* and 11/15 (73.3%) for *mpb*70 and for both markers. This was expected, as these samples were taken during consultations of symptomatic patients of the TTHALESS project. The relevant finding is the high sensitivity of both PCRs. For comparison, for the same patients the sensitivity of sputum testing was 77.8%, 62.5% and 88.9% for auramine staining, Z-N and GenXpert, respectively, and 83.3%, 33.3% and 66.7% for faeces (Supplementary file, Table S2). Our results thus support the sensitivity of sponge-based eDNA sampling for detecting active human TB cases.

**Figure 3.**
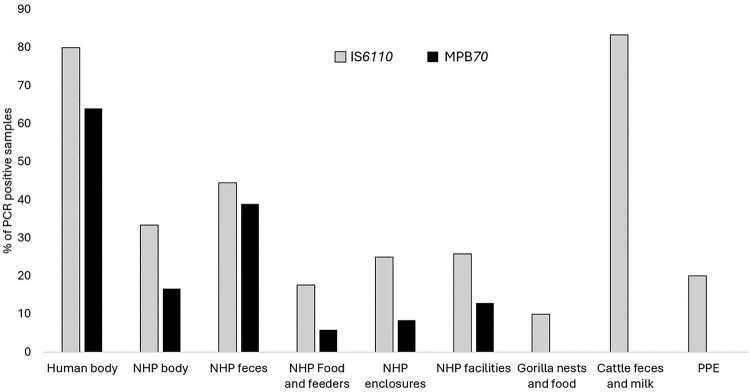
*Mycobacterium tuberculosis* complex (MTBC) DNA detection in samples taken on different substrates at the human – non-human primate (NHP) interface in eastern DRC. Test results for 172 environmental sponge eDNA samples taken on humans (n = 25), anesthetized NHPs (n = 11), NHP faeces (n = 19), from captive NHP food and feeders (n = 17), NHP enclosures (n = 36), outer NHP facilities (n = 31), wild gorilla nests and food (n = 20), cattle faeces and milk (n = 6), PPE (n = 5), and hospital surfaces (n = 2).

Regarding the sampling targets, Figure 3 shows the MTBC eDNA testing results by sampling substrate. The highest proportion of positive tests was found in samples taken on human body surfaces, followed by samples taken on NHP faeces. The lowest positivity rates were found for PPE and wild gorilla environment samples. Gorilla nest and food samples, cattle samples, and PPEs were only found positive for IS*6110*.

## Discussion

Using non-invasive eDNA sampling, we detected *M. tuberculosis* in the environment of free ranging Eastern Lowland (Grauer's) Gorillas, a nearby primate rehabilitation centre, and the local human hospital. Sponge samples collected on humans, free ranging gorillas and captive NHPs shared the same spoligotype pattern. These findings are strongly suggestive of epidemiological links between human and NHP TB.

It is known that TB can be fatal to NHPs since it represents a threat to laboratory primate communities [30]. However, natural infection is rare in wild great apes and has not been recorded in wild gorillas [23,31]. Table S1 presents an incomplete list of MTBC infections reported in captive and free-ranging African NHPs. Regarding great apes, all detections of *M. tuberculosis* (n = 6 reports) and *M. bovis* (n = 2) occurred in captive chimpanzees (n = 5) and gorillas (n = 3), with no peer-reviewed reports regarding bonobos and free-ranging gorillas.

We describe the first MTBC eDNA detection in free ranging gorillas. It is also the first report of MTBC contact in Eastern Lowland Gorillas and has therefore important implications for great ape conservation. Finding MTBC DNA in gorilla faeces was not unexpected given the high human TB incidence in DRC and given the fact that the sampled gorilla groups were habituated to human contact. Finding high rates of positivity in samples from the LPRC, including samples collected on chimpanzees or in chimpanzee facilities is also relevant, especially in case of translocations [22]. In Gombe National Park, Tanzania, 144 chimpanzee and 62 olive baboon faecal samples were screened for MTBC targeting the IS*6110* marker by PCR, all resulting in negative results [32]. For comparison, we found positivity to the same IS*6110* marker in 61 of 172 samples (35%) from our study sites. The potential impact of a chronic and debilitating *M. tuberculosis* infection on great ape fitness and survival remains unknown.

Five different spoligotype patterns were found in the samples tested. One (SIT26) was detected in three chimpanzee enclosures at LPRC, while a second one (SIT130) was found across sampling sites. The latter spoligotype pattern was detected on gorilla faeces, on humans in contact with Gorillas (KBNP rangers), on hospital patients, and in samples collected in the LPRC. This suggests links between human *M. tuberculosis* infection and *M. tuberculosis* detection in great apes or their environment, including free ranging gorillas and captive NHPs. It does also suggest epidemiological links between compartments, namely the national park, the local community, and the rehabilitation centre. Infections by spoligotype SIT130 are primarily associated with the LAM (Latin American-Mediterranean) family of *M. tuberculosis*, with a significant presence in Latin America, Europe, and Africa [29] (SITVIT2 website).

Spoligotype SIT26 (CAS1-Delhi) is most often identified on the Indian subcontinent with several detections in sub-Saharan Africa, too, but no prior records in DRC [29] (SITVIT2 website). This spoligotype may have been introduced to the sanctuary through an infected animal that had spent some time with humans after its capture, during which it became infected. We were surprised not to find *M. bovis* in this survey, since *M. bovis* infection has been reported in both great apes and other NHPs in Africa (Table S1) and locals are in contact with cattle and other susceptible livestock (Figure 1).

While eastern DRC is an extremely poor region where several human TB risk factors such as undernourishment, AIDS and other coinfections, alcohol abuse, and poor hygiene converge, TB is also highly prevalent in other neighbouring countries with great ape populations [7,33]. Thus, the risk of infection transmission from humans to apes in and around African protected areas or in rehabilitation centres needs to be considered, confirming earlier suspicions [24]. From a public health point of view, the risk posed to humans by infected great apes is negligible except for caretakers at rehabilitation centres and similar occupations. However, preventive measures such as masking should be compulsory for rescue centre and national park staff, especially for people with active TB, as well as for rehabilitation centre visitors and for gorilla tourism [34,35].

Our study has several limitations. First, DNA-based methods do not distinguish between viable and dead MTBC cells [13,36]. However, environmental sampling enabled us to study a challenging setting where direct access to some species would have been impossible. Culture-based techniques are not easy to run under the circumstances of war-torn South Kivu. We found positiveness to both markers in 7 captive NHP groups and not in the 5 remaining ones. If the detection lacked specificity we would have expected to find positive samples in all settings. Further, our eDNA detection targeted two sequences, the highly sensitive (multicopy) IS*6110* and the more specific but less sensitive (unicopy) *mpb*70, and all but one *mpb*70 positive samples were also IS*6110* positive. One third of the IS*6110* positive but *mpb*70 negative samples were confirmed by spoligotyping, FluoroType MTBDR or Vircell. Moreover, the recovered eDNA was of sufficient quality to assign half of the *mpb*70 positive samples to a spoligotype pattern.

Secondly, the sample sizes were not proportional between sampling sites, mainly due to differences in accessibility. As a result, 116 samples were collected at the LPRC, 39 samples in the KBNP, and only 23 samples in the Miti-Murhesa health area including Lwiro hospital. For future studies, it would be advisable to improve the sample size to ensure a more balanced distribution. Thirdly, it would also be desirable to include other animal species that may influence TB epidemiology in the study region. Specifically, future studies should include other free-ranging NHPs like Olive Baboons and Chimpanzees.

This is the first application of eDNA sponges to settings at the human-wildlife interface. Sponge-based MTBC eDNA detection is a cheap and convenient tool for MTBC detection in human dwellings or in healthcare facilities. For free ranging great ape populations, this method fits the need to couple syndromic surveillance with targeted diagnostic sampling to improve early outbreak detection and population impact assessment [37]. In conclusion, our results support epidemiological links between human and NHP TB in equatorial Africa and show that sponge-based sampling represents a useful tool for TB surveillance and risk assessment in challenging environments.

## Author contributions

EK: data curation, formal analysis, investigation, methodology, visualization, writing-original draft, writing-review and editing. LF: data curation, formal analysis, funding acquisition, investigation, validation, methodology, visualization, supervision, project administration, writing-original draft, writing-review and editing. MPS: data curation, formal analysis, investigation, validation, methodology, visualization, resources, writing-original draft, writing-review and editing. AP: conceptualization, data curation, formal analysis, investigation, validation, methodology, visualization, resources, supervision, project administration, writing-original draft, writing-review and editing. CH: data curation, investigation, methodology, writing-original draft, writing-review and editing. LH: data curation, investigation, methodology, validation, writing-original draft, writing-review and editing. BR: data curation, investigation, methodology, validation, writing-original draft, writing-review and editing. TGS: data curation, investigation, methodology, validation, writing-original draft, writing-review and editing. DK: data curation, writing-review and editing. AK: data curation, writing-review and editing. ZK: data curation, writing-review and editing. FBM: data curation, writing-review and editing. DB: data curation, writing-review and editing. PN: data curation, writing-review and editing. IVB: data curation, writing-review and editing. FLG: data curation, writing-review and editing. JF: funding acquisition, investigation, validation, methodology, resources, writing-original draft, writing-review and editing. LD: funding acquisition, investigation, validation, methodology, visualization, resources, supervision, project administration, writing-original draft, writing-review and editing. CG: conceptualization, funding acquisition, formal analysis, investigation, validation, methodology, visualization, resources, supervision, project administration, writing-original draft, writing-review and editing.

## Supplementary Material

Supplementary information_amended.docx

## Data Availability

The data that support the findings of this study are openly available in the ZENODO repository at doi: 10.5281/zenodo.17133952. Further supplementary information and data are available from the corresponding authors upon reasonable request (Alberto Perelló: albertoperellojimenez@gmail.com or Christian Gortázar: christian.gortazar@uclm.es).
