# Supplementary material for: Non-invasive environmental DNA sampling reveals tuberculosis risks at the human – Great Ape Interface in Africa
Source: Emerg Microbes Infect. 2026 Mar 23;15(1):2645874. doi: 10.1080/22221751.2026.2645874 (PMC13011094; doi:10.1080/22221751.2026.2645874)
Supplement: Supplementary information_amended.docx [file TEMI_A_2645874_SM6254.docx]

**Non-invasive environmental DNA sampling reveals tuberculosis risks at the human – great ape interface in Africa.**

**Sampling procedures.**

Body (Anesthetized NHPs, LPRC): Sponges were passed over the entire body surface of the animals during anesthesia; Gorilla Feces (KBNP): Sponges were passed over gorilla feces found in the forest; NHP Feces (LPRC): Sponges were passed over the feces of non-human primates (NHPs) at the LPRC; Gorillas' Leftover Food (KBNP): Sponges were passed over leftover food discarded by gorillas after eating; Leftover Food in Feeders (LPRC): Sponges were passed over the surface of feeders after NHPs had eaten in indoors at the LPRC; Indoor Leftover Food (LPRC): Sponges were passed over food residues outside the feeders in the indoor enclosures; Gorilla Nests (KBNP): Sponges were passed over the surface of gorilla nests in the forest of KBNP; Human Body Surfaces: Sponges were passed over the nose, mouth, face, hands, and clothing of humans; PPE (KBNP): Sponges were passed over off-body clothing items such as boots and work clothes of humans in KBNP; PPE (LPRC): Sponges were passed over off-body clothing items such as boots and work clothes of humans in LPRC; NHPs Indoor Surfaces (LPRC): Sponges were passed over various surfaces inside the enclosures of the NHPs; NHPs Outdoor Surfaces (LPRC): Sponges were passed over various surfaces in the LPRC outside of the enclosure of NHPs; Sputum/Soil Samples (LPRC): Sponges were passed over the soil in the outdoor areas of the LPRC where sputum was present.

Tables

Table S1. Incomplete list of reports on *Mycobacterium tuberculosis* complex infections in African non-human primates (NHPs), including great apes (darker grey) and other NHPs (lighter grey).

| **NHP species** | **Agent** | **Setting** | **Reference** |
| --- | --- | --- | --- |
| Vervet monkey *Chlorocebus pygerethrus* | *M. bovis* | Free range with contact to cattle | [38] |
| Vervet monkey *Chlorocebus pygerethrus* | *M. tuberculosis* | Captive | [39] |
| Patas monkey *Erythrocebus patas* | *M. tuberculosis* | Captive | [39] |
| Yellow baboon *Papio cynocephalus* | *M. bovis* | Free range with contact to cattle | [38] |
| Yellow baboon *Papio cynocephalus* | *M. bovis* | Free range with contact to cattle | [40] |
| Chacma baboon *Papio ursinus* | *M. bovis* | Free range | [41] |
| Chacma baboon *Papio ursinus* | *M. bovis* | Free range with contact to cattle | [42] |
| Chacma baboon *Papio ursinus* | *M. tuberculosis* | Free range and captive | [39] |
| Marmoset *Callithrix jacchus* | *M. tuberculosis* | Captive | [43] |
| Chimpanzee *Pan troglodytes* | *M. bovis* | Captive | [33] |
| Chimpanzee *Pan troglodytes* | *M. tuberculosis* | Captive | [44] |
| Chimpanzee *Pan troglodytes* | *M. tuberculosis* | Captive | [45] |
| Chimpanzee *Pan troglodytes* | *M. tuberculosis* | Captive | [46] |
| Chimpanzee *Pan troglodytes* | Chimpanzee bacillus or *M. africanum* | Free range | [23] |
| Chimpanzee *Pan troglodytes* | *M. tuberculosis* | Captive | [47] |
| Gorilla *Gorilla gorilla* | *M. bovis* | Captive | [48] |
| Gorilla *Gorilla gorilla* | *M. tuberculosis* | Captive | [49] |
| Gorilla *Gorilla gorilla* | *M. tuberculosis* | Captive | [39] |

**Table S2.** Additional TB testing of patients enrolled in the TTHALESS project. Human body surface sponge PCR results (this study) in samples taken at LH compared to results obtained in the TTHALESS project for the same individuals. Patient NKPA157 was sampled on two occasions.


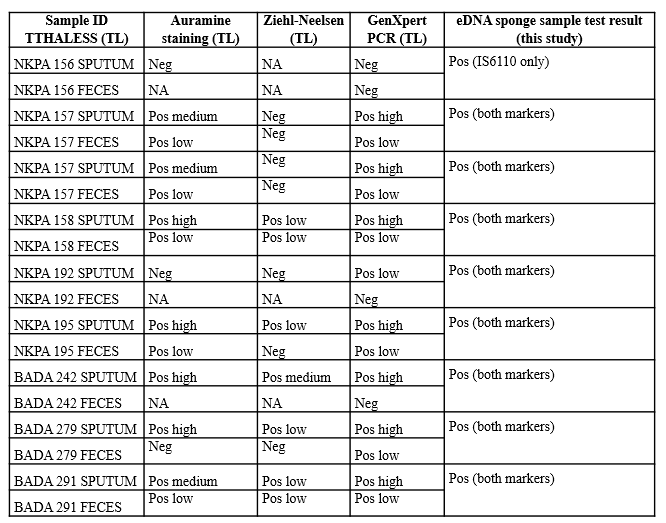
—Pos = positive; Neg = negative—
